# MicroRNA-155 Participates in Smoke-Inhalation-Induced Acute Lung Injury through Inhibition of SOCS-1

**DOI:** 10.3390/molecules25051022

**Published:** 2020-02-25

**Authors:** Yue Zhang, Yifang Xie, Leifang Zhang, Hang Zhao

**Affiliations:** 1 Collaborative Innovation Center of Yangtze River Delta Region Green Pharmaceuticals, Zhejiang University of Technology, Hangzhou 310014, China; spiritak@163.com (Y.Z.); xieyf0718@163.com (Y.X.); 2 Zhejiang Provincial Engineering Technology Research Center of Marine Biomedical Products, School of Food and Pharmacy, Zhejiang Ocean University, Zhoushan 316022, China; zhangleifang1986@163.com

**Keywords:** acute lung injury, neutrophils, microRNA-155, suppressor of cytokine signaling-1

## Abstract

Smoke inhalation causes acute lung injury (ALI), a severe clinical disease with high mortality. Accumulating evidence indicates that microRNA-155 (miR-155) and suppressor of cytokine signaling 1 (SOCS-1), as mediators of inflammatory response, are involved in the pathogenesis of ALI. In this paper, we explored the proinflammatory mechanism of miR-155 in smoke-inhalation-induced ALI. Our data revealed that smoke inhalation induces miR-155 expression, and miR-155 knockout (KO) significantly ameliorates smoke-inhalation-induced lung injury in mice. Neutrophil infiltration and myeloperoxidase (MPO), macrophage inflammatory protein 2 (MIP-2) and keratinocyte chemoattractant (KC) expressions were decreased in miR-155^–/–^ mice after smoke inhalation as well. Real-time RT-PCR and immunoblotting results showed that SOCS-1 level was remarkably increased in miR-155^–/–^ mice after smoke exposure. Furthermore, the experiments performed in isolated miR-155 KO pulmonary neutrophils demonstrated that the lack of SOCS-1 enhanced inflammatory cytokines (MIP-2 and KC) secretion in response to smoke stimulation. In conclusion, smoke induces increased expression of miR-155, and miR-155 is involved in inflammatory response to smoke-inhalation-induced lung injury by inhibiting the expression of SOCS-1.

## 1. Introduction

Burns are the fourth most common type of injury in the world, ranking behind traffic accidents, falls and intentional injuries. Millions of people suffer from burn injuries globally every year, with nearly 300,000 succumbing to mortality [1]. Although the survival rate from burn injuries has increased in recent years with the development of effective fluid resuscitation management and early surgical excision of burned tissue, the mortality from burn injuries is still high. Acute lung injury (ALI) is a type of respiratory failure characterized by diffuse, acute pulmonary inflammation. Smoke-inhalation-induced acute lung injury (SI-ALI), acute respiratory distress syndrome (SI-ARDS), or even serious respiratory failure contribute to ninety percent of burn-related mortality [2]. Epidemiological investigations have shown that the in-hospital mortality of SI-ALI reaches 26% [2]. However, the molecular mechanisms involved in the pathogenesis of smoke-inhalation-induced acute lung injury are poorly defined [3,4]. In the pathological process of SI-ALI, it is generally believed that inflammatory cells and release of inflammatory mediators, especially neutrophils and macrophages, are mandatory [3,4,5]. The accumulation of neutrophils in the lung microvasculature, interstitium and alveolar space is a key feature of SI-ALI/ARDS [3,5]. Neutrophils are the first leukocytes to be recruited to sites of inflammation in response to chemotactic factors released by activated macrophages and pulmonary epithelial and endothelial cells. Excessive recruitment and activation of neutrophils can lead to bystander tissue damage and further loss of lung function. Therefore neutrophils and their associated chemotactic factors in SI-ALI/ARDS are thought to significantly contribute to the disease progression. Despite decades of research, a number of pharmacologic therapies for ALI have failed clinical trials due to weak efficacy or potential harm [6]. Hence, further exploration of signaling pathways in SI-ALI is crucial for not only advancing our understanding of the pathogenesis of this life-threatening disease but also the development of novel therapeutic intervention.

Suppressor of cytokine signaling 1 (SOCS-1), also known as STAT-induced STAT inhibitor 1 (SSI-1) and JAB, is a member of cytokine signaling inhibitor family, which regulate the production of several cytokines associated with inflammation [7]. Several studies indicate that SOCS-1 is an important regulator in pulmonary inflammation [7,8]. Earlier experiments show that SOCS-1 restores majority of interleukin-1β (IL-1β)-mediated cellular damage during hyperoxic lung injury [8,9]. Exogenous SOCS-1 adenovirus (Ad-SOCS-1) administration exhibits protective effect against bleomycin-induced pulmonary inflammation and fibrosis as well as hyperoxic acute lung injury [7,10]. Our recent report indicates that SOCS-1 is a critical regulator against SI-ALI through an anti-inflammatory mechanism. SOCS-1 relieves smoke-inhalation-induced pulmonary inflammation and injury by inhibiting NALP3 inflammasome formation [4].

MicroRNAs (miRNAs) are endogenous small noncoding RNA molecules that function in post-transcriptional regulation of gene expression via targeting mRNAs for promoting mRNA cleavage or repressing protein translation [11,12]. Several studies show that miRNAs serve as regulators in the pathogenesis of ALI [13,14]. Among large miRNA family, microRNA-155 (miR-155) plays a crucial role in various biological processes, including hematopoiesis, inflammation, cancers, innate immunity and acquired immunity [15]. It is found that miR-155 is induced in macrophages and monocytes by various inflammatory mediators, for instance, lipopolysaccharide (LPS), interferon-β (IFN-β) or allergen [16,17]. Previous studies demonstrated that upregulated miR-155 promotes inflammation in multiple-stimuli-induced ALI, including staphylococcal enterotoxin B [18], LPS [19] and high-tidal-volume ventilation [14]. Moreover, it is demonstrated that antisense therapy against miR-155 can enhance the recovery of ALI in mice [20]. Importantly, miR-155 has a strong correlation with SOCS-1 in various pathological conditions. MiR-155 is identified as a proinflammatory factor which targets and downregulates SOCS-1 [21]. Luciferase reporter assay demonstrates that SOCS-1 is directly targeted by miR-155 on the 3′-untranslated region (3′-UTR) [19]. Some studies indicate that miR-155 can modulate immune response by suppressing the expression of SOCS-1 in multiple inflammatory models including LPS-induced ALI [19,22,23]. Those studies strongly suggest that miR-155 might be a potential target for the therapy of ALI. However, the specific mechanism of miR-155 and the correlation with SOCS-1 in the pathogenesis of SI-ALI remains unclear.

Here, we hypothesized that miR-155 is involved in inflammatory response in SI-ALI and that this process may be related to its suppression effect of SOCS-1 expression. To test this hypothesis, miR-155 KO (miR-155^–/–^) mice and isolated neutrophils were used to ascertain whether miR-155 exaggerates lung injury after smoke inhalation. We also silenced SOCS-1 in miR-155^–/–^ neutrophils and measured related inflammatory cytokines levels after smoke exposure to further investigate the correlation between miR-155 and SOCS-1 in SI-ALI.

## 2. Results

### 2.1. MiR-155 Expression Increased after Smoke Inhalation

**Figure 1:**
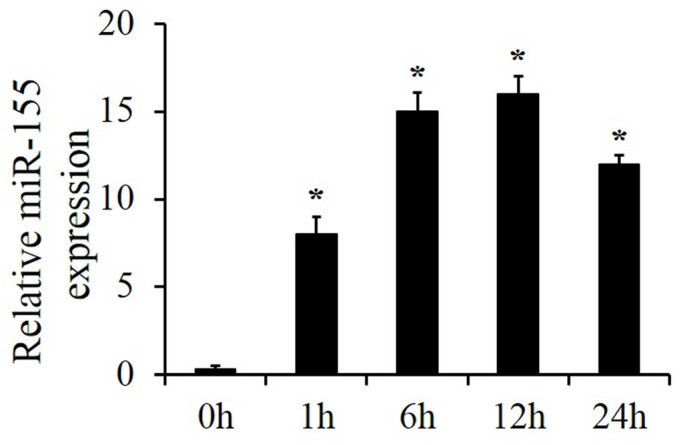
MiR-155 expression increased in smoke-induced acute lung injury (ALI). ALI was induced in mice by smoke. After recovery for the indicated time periods (0, 1, 6, 12 and 24 h), lung RNA levels of miR-155 were analyzed by real-time RT-PCR. *n* = 4 samples per group. *p < 0.05 vs. 0 h.

Wild-type (WT) mice were treated with smoke for 15 min and sacrificed after indicated time periods (0, 1, 6, 12 and 24 h). Real-time RT-PCR results showed that miR-155 level in lung tissue increased significantly and culminated at around 12 h after smoke inhalation (Figure 1). Thus, we speculated that lung injury might be associated with increased miR-155 expression and selected 12 h as the optimal smoke inhalation time period for the following animal experiments.

### 2.2. Absence of Mir-155 Relieved Smoke-Inhalation-Induced ALI

**Figure 2:**
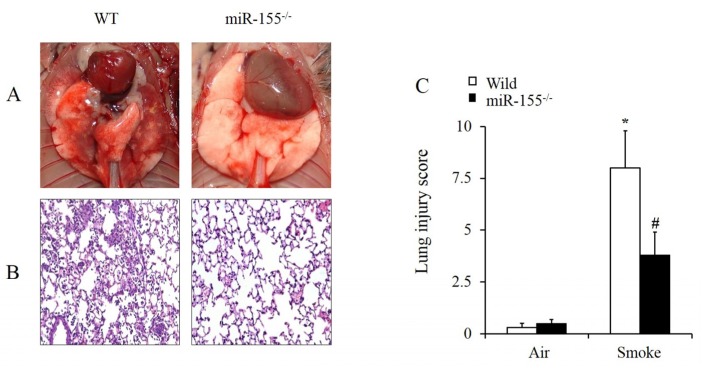
MiR-155 deficiency was associated with decreased smoke-induced lung injury. (**A**) At 12 h after smoke, pathological observation of the lungs in mice was performed. (**B**) Lung sections were stained with H&E, original magnification 200×. (**C**) Lung injury scores were measured and calculated. *n* = 4 samples per group. *p < 0.05 vs. wild-type (WT) mice treated with air. #*p* < 0.05 vs. WT mice treated with smoke.

WT and miR-155^–/–^ mice were treated with smoke for 15 min then sacrificed 12 h later to observe the lung pathological degree of change for each group. As shown in Figure 2A, WT mice exhibited typical lung injury symptoms after smoke inhalation: lung tissues turned dark red with extensive exudation, diffuse hyperemia and edema. In contrast, miR-155^–/–^ mice had little injury after smoke inhalation: lung tissues remained pink, and no obvious bleeding, exudation or edema were observed. The results show that miR-155 KO significantly attenuates lung tissue damage caused by smoke inhalation.

Then, H&E staining was performed for further assessments of degree of lung injury in WT and miR-155^–/–^ mice. Lung sections were stained with H&E and observed under microscope. In WT mice, lung sections indicated immune cells infiltration in pulmonary interstitial and edema in alveolar epithelial cells. Compared to WT mice, infiltration of neutrophils and monocytes and thickening of alveolar septum were reduced remarkably in miR-155^–/–^ mice (Figure 2B). Histological examinations showed that the loss of miR-155 protected mice from excessive inflammatory response. Accordingly, we established lung injury scores to estimate degree of injury. Parameters include alveolar hemorrhage, alveolar inflammatory cells infiltration and alveolar septal congestion. Quantitative scoring of the severity of histological lung injury showed that the lung injury score was significantly lower in miR-155^–/–^ mice than in WT mice after smoke inhalation (WT: 8 ± 1.8, miR-155^–/–^: 3.8 ± 1.1). WT and miR-155^–/–^ mice treated with air were also taken into account and the scores suggested that no obvious lung injury was observed in both groups (Figure 2C). Altogether, these results reveal that absence of miR-155 significantly alleviates smoke-inhalation-induced acute lung injury in mice.

### 2.3. MiR-155 KO Reduced Neutrophil Aggregation and Inflammatory Cytokines Release in the Lung

**Figure 3:**
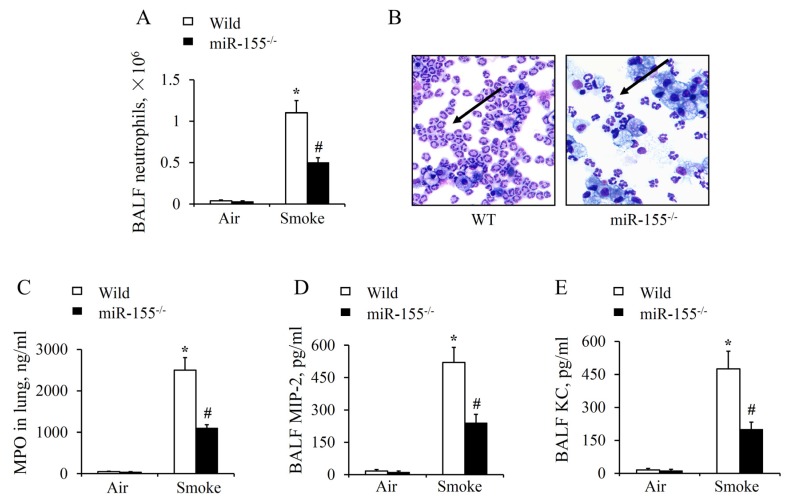
MiR-155 absence attenuated neutrophil activation and accumulation. Bronchoalveolar lavage fluid (BALF) and lung tissue were harvested at 12 h after 15 min of smoke exposure. (**A**) Cell counts of BALF neutrophils was determined. (**B**) Diff-Quick-stained cytospins of bronchoalveolar lavage neutrophils after smoke inhalation, original magnification 400×. (**C**–**E**) The myeloperoxidase (MPO) level in lung tissue and macrophage inflammatory protein 2 (MIP-2) and keratinocyte chemoattractant (KC) proteins in BALF were measured by ELISA. *n* = 4 mice per group. *p < 0.05 vs. WT mice treated with air. #*p* < 0.05 vs. WT mice treated with smoke.

To further demonstrate that miR-155 exaggerated smoke-inhalation-induced lung injury, a series of experiments were performed on the molecular and cellular levels. Lung tissues and bronchoalveolar lavage fluid (BALF) were collected from WT and miR-155^–/–^ mice for examination. Compared with the groups treated with air, we found that there was a significant increase in neutrophil count (NEUT) after smoke stimulation (Figure 3A). While neutrophil accumulation in BALF of miR-155^–/–^ was significantly less intense in comparison with WT after smoke (Figure 3B). In the meanwhile, myeloperoxidase (MPO) level in the lung tissues, which reflected the functional status and activity of neutrophil, rose markedly as well (Figure 3C). Both results suggest that neutrophils were largely activated and recruited into the lung in response to smoke exposure. By contrast, depletion of miR-155 observably reduced neutrophil recruitment and MPO level in injured lungs. Similarly, ELISA results showed that macrophage inflammatory protein 2 (MIP-2) and keratinocyte chemoattractant (KC) production were greatly decreased in miR-155^–/–^ mice (Figure 3D,E). Consequently, these results suggest that chemotactic factors secretion and neutrophil recruitment are held back by miR-155^–/–^ deficiency.

### 2.4. MiR-155 Deficiency Made SOCS-1 Expression Increase in Both Lung Tissues and Neutrophils after Smoke Exposure

**Figure 4:**
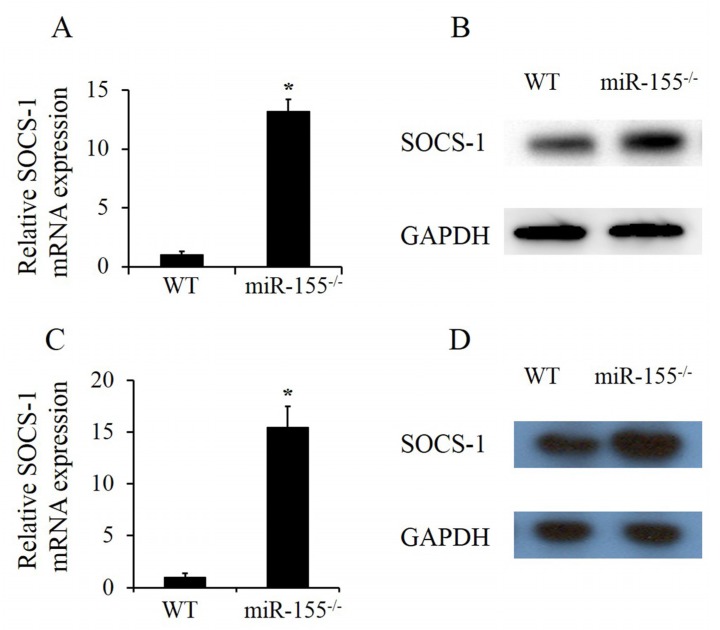
MiR-155 suppressed suppressor of cytokine signaling 1 (SOCS-1) in lung tissues and neutrophils. (**A**,**B**) MiR-155^–/–^ mice were smoke-exposed for 15 min and sacrificed after 12 h, following which SOCS-1 mRNA and protein level of whole lung homogenates were measured. Neutrophils were isolated from lung tissues after 12 h following smoke exposure, and isolated neutrophils were treated with smoke for 30 min. (**C**,**D**) After 24 h of culture, SOCS-1 mRNA and protein level of isolated neutrophils were analyzed. *n* = 4 mice per group. *p < 0.05 vs. WT mice treated with smoke.

Our previous studies found that smoke promotes inflammation by inhibiting SOCS-1 expression [4]. To determine the relationship between miR-155 and SOCS-1 in smoke-inhalation-induced ALI, SOCS-1 mRNA and protein expressions in smoke-exposed lung homogenates were assayed in both wild-type and miR-155^–/–^ mice. RT-PCR and immunoblotting results showed that both SOCS-1 mRNA (Figure 4A) and protein (Figure 4B) levels increased remarkably in lungs of miR-155^–/–^ mice compared to the levels in wild-type ones. SOCS-1 expression in pulmonary neutrophils was analyzed as well. After 12 h following 15 min of smoke exposure, neutrophils which had been recruited were isolated from lung tissues. Afterwards, isolated neutrophils were stimulated by smoke for 30 min then cultured for 24 h. Consistently, SOCS-1 mRNA and protein expressions were highly upregulated in miR-155^–/–^ neutrophils (Figure 4C,D). Together, these results demonstrate that the ability of smoke to dampen SOCS-1 expression is stunted without miR-155 in both lung tissues and neutrophils.

### 2.5. SOCS-1 Silencing Enhanced Smoke-Induced Inflammatory Response in Mir-155^–/–^ Neutrophils

**Figure 5:**
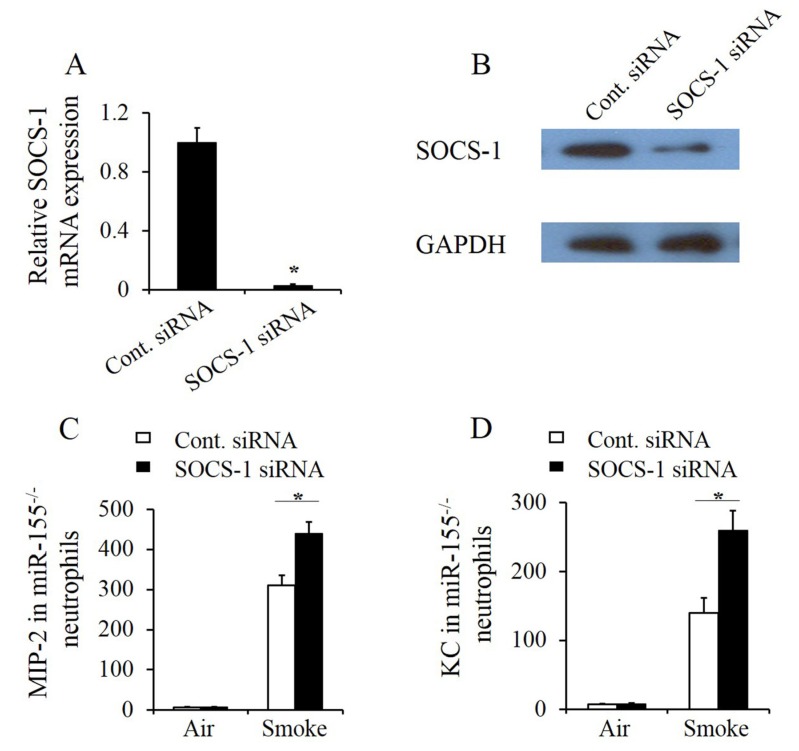
MiR-155 targeted SOCS-1 to regulate inflammatory response in neutrophils. Whole lung neutrophils were isolated from miR-155^–/–^ mice 12 h after smoke inhalation as described in Section 4, and isolated neutrophils (2 × 10^6^/mL) were transfected with SOCS-1 siRNA or control siRNA. (**A**,**B**) After 24 h of culture, SOCS-1 mRNA and protein level in neutrophils were measured. *n* = 4 mice per group. *p < 0.05 vs. neutrophils transfected with control siRNA. Lung neutrophils were isolated from miR-155^–/–^ mice 12 h after 15 min of smoke inhalation. Isolated neutrophils were transfected with SOCS-1 siRNA or control siRNA. (**C**,**D**) After 24 h of transfected cell culture following smoke treatment of 30 min, the supernatant medium was collected and used to measure MIP-2 and KC protein by ELISA 24 h after smoke exposure. *n* = 4 mice per group. *p < 0.05.

To further investigate the role of SOCS-1 in miR-155 regulated pulmonary inflammation, SOCS-1-silenced miR-155^–/–^ mouse pulmonary neutrophils were constructed. Neutrophils were extracted from miR-155^–/–^ lung tissues in mice after 12 h following smoke exposure 15 min. Thereafter, SOCS-1 silencing was performed by small interfering RNA (siRNA) transfection and confirmed by real-time RT-PCR (Figure 5A) and immunoblotting (Figure 5B). SOCS-1-silenced miR-155^–/–^ neutrophils then were treated with or without smoke for 30 min. After 24 h of cultivation, the culture supernatant was collected and examined. ELISA results exhibited that MIP-2 (Figure 5C) and KC (Figure 5D) levels were increased in SOCS-1-silenced neutrophils after smoke stimulation. Thus, the results consolidate that miR-155 suppresses SOCS-1 to exaggerate inflammatory response in neutrophils.

## 3. Discussion

**Figure 6:**
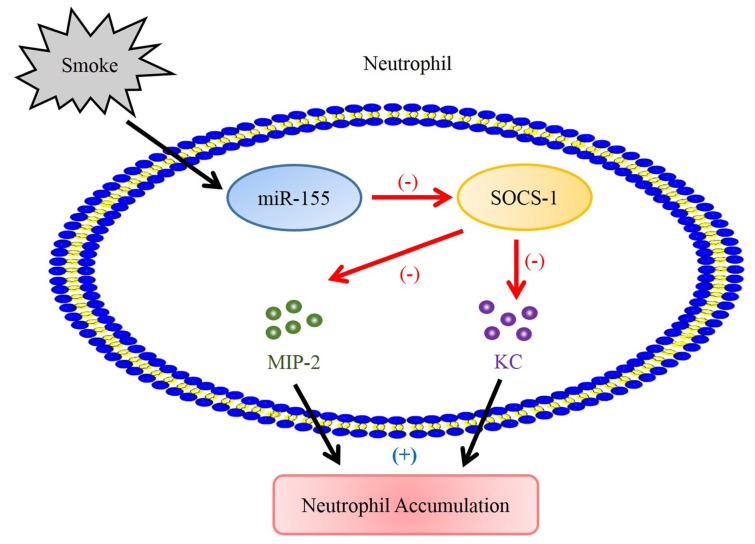
Model of miR-155-modulated neutrophil activation and recruitment through suppression of SOCS-1 smoke-induced in pulmonary neutrophil. Smoke-elevated miR-155 expression suppresses SOCS-1, which is a negative regulator of neutrophil chemoattractants. Subsequently, upregulated MIP-2 and KC function cooperatively to promote neutrophil accumulation in the lung.

Herein, we investigated the roles and relationships of miR-155 and SOCS-1 in pathogenesis of smoke-inhalation-induced ALI. The results indicate that miR-155 exhibits proinflammatory effect in smoke-inhalation-induced acute lung injury. MiR-155 mediates MPO and neutrophil chemoattractants production by suppressing SOCS-1 to exaggerate lung injury after smoke exposure (Figure 6).

As a newly discovered gene regulator, miR-155 has drawn increasing attention for possible mediation of ALI progression and is reported to be involved in regulating proinflammatory factors in multifactor-induced ALI [18,19,23,24]. Correspondingly, inhibition or KO of miR-155 shows protective effect on exaggerated inflammatory responses to reduce tissues or cells damage [25,26]. Consistently, we showed that miR-155 depletion relieved lung tissue exudation, hyperemia, edema and neutrophil infiltration obviously and reduced inflammatory cytokines (MPO, MIP-2 and KC) secretion in smoke-inhalation-induced ALI. Importantly, we demonstrated for the first time that miR-155 participates in the pathogenesis of smoke-inhalation-induced ALI as a proinflammation mediator and might be a feasible therapeutic target in treatment. Additionally, it is reported that upregulated miR-155 also exhibits suppressive effects on apoptosis of specific activated inflammatory cells to maintain their critical functions in immune response [27]. MiR-155 also prevents apoptosis in macrophages induced by LPS [28]. Although this phenomenon might be helpful for the host defense against the invasion of pathogens, prolonged life span of inflammatory cells might contribute to excessive inflammatory responses which cause tissue injury. There is evidence that neutrophils from the lungs of patients with ALI are much less likely to become apoptotic [29]. Promoting neutrophil apoptosis is also conducive to the alleviation of ALI [30,31]. Therefore, the prevention of neutrophil apoptosis might be another pathway for miR-155 to exert proinflammatory effect in smoke-inhalation-induced ALI. In the previous work, we showed that IFN-β, which is an inducer of miR-155, was associated with impaired apoptosis of neutrophils in lungs [32]. However, more compact evidence is needed to manifest whether neutrophil apoptosis is attenuated by miR-155 in the pathogenesis of smoke-induced lung injury.

In our previous study, we found that adenoviral gene transfer of SOCS-1 improved the survival of smoke-exposed mice and SOCS-1 dampened NALP3 inflammasome assembly in smoked mouse pulmonary macrophages in vitro [4]. As shown in our results and other studies, miR-155 KO or inhibition results in upregulation of SOCS-1, indicating that miR-155 is a negative regulator of SOCS-1 [19,22]. Likewise, in the present work, SOCS-1 silencing partially reversed the anti-inflammatory effects in miR-155^–/–^ mouse pulmonary neutrophils, suggesting that miR-155 modulates smoke-inhalation-induced ALI through SOCS-1 suppression in neutrophils. It has also been pointed out that SOCS-1 exerts cytoprotection against apoptosis initiated by tumor necrosis factor-*α*, IFN-*γ* and apoptosis-inducing chemicals in tissue injury [9]. SOCS-1 overexpression rescues alveolar epithelial cells from apoptosis in hyperoxia-induced ALI through apoptosis signal-regulating kinase 1 (ASK-1) degradation [8,9]. Consistently, our previous study demonstrates that overexpression of SOCS-1 protects alveolar epithelial cells from reduction of cellular viability and inhibits apoptosis induced by smoke via degradation of ASK-1 [33]. Collectively, an inference can be drawn that miR-155 may accentuate tissue damage through promoting alveolar epithelial cells apoptosis via suppression of SOCS-1-induecd ASK-1 degradation in smoke-inhalation-induced ALI.

Neutrophil accumulation in the lung is a critical step in the early stages of ALI. MIP-2 and KC possess powerful neutrophil chemotaxis. These chemoattractants are essential contributors to neutrophil activation and migration [34]. It has been found that KC and MIP-2 function cooperatively in the process of neutrophil infiltration [34]. KC release is responsible for inducing neutrophils in circulation at first. Subsequently, MIP-2 serves as a more potent signal to promote neutrophils adhesion to endothelial cells [35]. The suppression of MIP-2 and KC production and their receptors expression lead to attenuation of neutrophil accumulation in endotoxin-induced ALI [36,37]. Research shows that miR-155 modulates leukocyte recruitment via suppression of SOCS-1 [38]. In addition, SOCS-1 suppression significantly enhances MIP-2 production and neutrophil recruitment in ALI induced by LPS [39]. Similarly, in the current work, SOCS-1 silencing notably elevates MIP-2 and KC levels in neutrophils, indicating that SOCS-1 attenuates smoke-induced inflammation by suppressing neutrophil activation and recruitment. Previously, it was revealed that the expression of KC and MIP-2 can be stimulated by IL-1β through nuclear factor-κB signaling pathway [40]. In our previous work, we also demonstrated that IL-1β is a central mediator in the production of neutrophil chemoattractants in noninfectious lung inflammation [41]. Then, as mentioned above, our previous study revealed that NALP3 inflammasome assembly, which participates in IL-1β activation, is diminished by SOCS-1 in smoke-exposed pulmonary macrophages [4]. Hence, we inferred that SOCS-1 might regulate chemoattractants production in a similar mechanism through repressing IL-1β activation in pulmonary neutrophils. Furthermore, it was worth noting that chemoattractants expression in smoked neutrophils without SOCS-1 silencing remained markedly higher than groups treated with air, suggesting that there might be multiple pathways mediating activation and recruitment of neutrophils in smoke-inhalation-induced ALI. Recent studies indicate that various pathways including focal adhesion kinase (FAK), extracellular regulated protein kinases (ERK), p38 and G protein-coupled receptor kinase 2 (GRK2) are associated with neutrophil recruitment in the pathogenesis of ALI as well [36,42]. We will conduct further research on this issue in the future.

In summary, the present work shows that miR-155 significantly promotes smoke-inhalation-induced ALI through inhibition of SOCS-1. SOCS-1 serves as a critical negative regulator of neutrophil recruitment in smoke-inhalation-induced ALI. These results may lead to more in-depth understanding of the regulatory mechanism in smoke-inhalation-induced ALI and provide some new ideas for the development of therapeutic treatment against this lethal disease.

## 4. Materials and Methods

### 4.1. Animals

C57BL/6 male mice aged 8 weeks were purchased from the Zhejiang Academy of Medical Sciences. MiR-155-KO mice were obtained from Jackson Laboratories. All animals were maintained under specific-pathogen-free conditions. All procedures involving mice were approved and monitored by the animal experiment center of Zhejiang University of Technology (Approval No. 20190107005).

### 4.2. Smoke Inhalation

Smoke inhalation was performed as previously described [4]. Mice were placed in the smoke chamber after intraperitoneal anesthesia induced by 5% chloral hydrate 0.01 mL/g (Shanghai Aladdin Biochemical Technology Co., Ltd., Shanghai, China). Smoke was produced from 300 mg of smoldering cotton heated to 400 °C for 15 min. The smoke chamber was equipped with a simple thermal controller which kept the temperature from exceeding 40 °C to prevent thermal airway injury during smoke exposure. All mice were put back in the cages for recovery and free access to food and water was allowed during the experiment.

### 4.3. Bronchoalveolar Lavage Fluid

Mice were sacrificed 12 h after smoke exposure. The trachea was exposed through a midline incision and cannulated with a sterile 22-gauge needle. Cold PBS was instilled and collected through the incised trachea for four times, 0.5 mL each time. A total volume of 1.8 mL BALF was retrieved per mouse. After centrifugation, the supernatants of each lavage fluid sample were stored at −80 °C until use. Total cell numbers in BALF of each sample were counted in a hemocytometer. Determination of BALF neutrophil counts were performed on cytospin preparations stained with a Diff-Quick staining kit (IMEB, San Marcos, CA, USA).

### 4.4. Real-Time RT-PCR

RNA was extracted from lungs or isolated neutrophils using TRIzol Reagent (Invitrogen, Carlsbad, CA, USA) following the manufacturer’s instruction. One microgram of total RNA was reverse transcribed to cDNA using SuperScript II RNase H^–^ Reverse Transcriptase (Invitrogen, Carlsbad, CA, USA). PCR was performed using SYBR Green PCR Master Mix (Thermo Fisher Scientific, Waltham, MA, USA) with the following primers: SOCS-1, 5′-ACCTTCTTGGTGCGCGAC-3′, 5′-GGGCCCGAAGCCATCTT-3′; miR-155, 5′-CTCGTGTTAATGCTAATTGTGA-3′, 5′-GTGCAGGGTCCGAGGT-3′.

### 4.5. Lung Histology

Mice were euthanized and lungs were collected twelve hours after smoke inhalation. Each lung was fixed in 10% formalin overnight, followed by paraffin embedding. The fixed lungs were then sectioned at 4 μm of thickness. The tissue slices were deparaffinized in ethanol, rehydrated, and stained with hematoxylin and eosin.

### 4.6. Lung Injury Score

Semiquantitative assessment of lung injury was performed according to methods previously described [32]. At least five fields were analyzed for each lung slice.

### 4.7. ELISA and Myeloperoxidase Assay

MIP-2 and KC levels in BALFs were measured using mouse ELISA assay kits (R&D Systems, Minneapolis, MN, USA). After BALFs were collected, the whole lung homogenates were prepared. MPO level in lung tissues was analyzed with a mouse MPO ELISA kit (Cell Sciences, Canton, MA, USA) according to the manufacturer’s protocol.

### 4.8. Immunoblotting

Extracts from lung tissues or neutrophils were electrophoresed on SDS-PAGE gels (Bio-rad, Hercules, CA, USA) and transferred to polyvinylidene difluoride membranes (Millipore, Billerica, MA, USA). The membranes were washed in 1× PBS Tween-20 for 20 min and blocked with 5% skim milk at room temperature for 2 h. Then the membranes were incubated at 4 °C overnight with SOCS-1 antibody (Abcam, Cambridge, MA, USA) or GAPDH antibody in tris-buffered saline with Tween-20 (TBST) containing 5% skim milk under gentle shaking. After 20 min washing with 1× PBS Tween-20, the membranes were incubated with the horseradish-peroxidase-coupled secondary antibody (Abcam, Cambridge, MA, USA) in TBST containing 3% skim milk at room temperature for 2 h. Proteins were detected using ECL chemiluminescence (GE Healthcare, Pittsburgh, PA, USA).

### 4.9. Preparation of Neutrophils from Mouse Lung

After anesthesia, the lungs were flushed in situ with 5 mL PBS through heart cannulation to remove the intravascular blood pool. Lung tissues were minced and digested with 200 μg/mL collagenase D and 40 μg/mL DNase I (both from Roche) in 10 mL RPMI 1640 medium at 37 °C for 1 h on a shaker. Subsequently, the enzymatically digested lung tissues were filtered through a stainless steel mesh. Annexin V microbeads were used to remove apoptotic cells, following the manufacturer’s instructions (Miltenyi Biotech, Bergisch Galdbach, Germany). Viable neutrophils were then prepared using the neutrophil isolation kit (Miltenyi Biotech, Bergisch Galdbach, Germany) and resuspended in RPMI 1640 medium containing 10% fetal calf serum and antibiotics.

### 4.10. Lung Neutrophils Smoke Exposure

Whole lung neutrophils were isolated from mice 12 h after smoke inhalation, and isolated neutrophils (2 × 10^6^/mL) were cultured in six-well plates. During smoke exposure, the temperature in the smoke chamber was maintained at 37 °C. The smoke was introduced to the cells at a flow rate 25 mL/min using an air sampling pump for 30 min. After smoke exposure, the cells were washed with 1× PBS, and incubated in fresh culture medium with 10% fetal calf serum with 5% CO_2_ in an incubator at 37 °C for 24 h. Cells were collected for subsequent experiments.

### 4.11. Knockdown of SOCS-1

The sequences of SOCS1 siRNA and control RNA were described previously [43]. The SOCS-1 siRNA was 5′-CUACCUGAGUUCCUUCCCCTT-3′ (sense) and 5′-GGGGAAGGAACUCAGGUAGTT-3′ (antisense). The control siRNA sequences were 5′-UUCUCCGAACGUGUCACGUTT-3′ (sense) and 5′-ACGUGACACGUUCGGAGAATT-3′ (antisense). siRNA duplexes were transfected into lung neutrophils at a final concentration of 30 nM by lipofectamine 2000 following the manufacturer’s instructions (Invitrogen, Carlsbad, CA, USA). Cells were incubated with siRNA for 24 h. The transfected cells were stimulated with smoke for 30 min. Twenty-four hours after smoke exposures, the supernatant medium was collected and used to measure MIP-2 and KC protein by ELISA.

### 4.12. Statistical Analysis

Data are expressed as mean ± SEM. A one-way analysis of variance followed by the Bonferroni test were used for multiple groups. When comparing between two groups, a Student’s *t*-test was used.
